# Relationship between 233 colorectal cancer risk loci and survival in 1926 patients with advanced disease

**DOI:** 10.1038/s44276-023-00003-z

**Published:** 2023-06-22

**Authors:** Christopher Wills, Amy Houseman, Katie Watts, Timothy S. Maughan, David Fisher, Richard S. Houlston, Hannah D. West, Valentina Escott-Price, Jeremy P. Cheadle

**Affiliations:** 1 Division of Cancer and Genetics, School of Medicine, Cardiff University, Heath Park, Cardiff, CF14 4XN UK; 2 CRUK/MRC Oxford Institute for Radiation Oncology, University of Oxford, Roosevelt Drive, Oxford, OX3 7DQ UK; 3 MRC Clinical Trials Unit, University College of London, 125 Kingsway, London, WC2B 6NH UK; 4 Division of Genetics and Epidemiology, The Institute of Cancer Research, London, SW7 3RP UK; 5 Division of Psychological Medicine and Clinical Neurosciences, School of Medicine, Cardiff University, Hadyn Ellis Building, Maindy Road, Cardiff, CF24 4HQ UK

## Abstract

**Background:**

Genome, transcriptome and methylome-wide association studies have identified single-nucleotide polymorphisms (SNPs) or genes at 258 loci associated with colorectal cancer (CRC) risk. We studied the relationship between these and patient outcome.

**Methods:**

We studied 1926 unrelated patients with advanced CRC from COIN and COIN-B. Of 205 CRC-risk SNPs, 19 were directly genotyped and 162 were imputed, and of 53 risk genes, 52 were tested. An additive Cox model for overall survival was adjusted for known prognostic factors. For nominally significant SNPs or genes, we considered a recessive model with a Bonferroni corrected threshold of *P* = 2.1 × 10^−4^. We examined SNPs as expression quantitative trait loci (eQTL) and the relationship between gene expression in colorectal tumours and survival in 597 unrelated patients.

**Results:**

Eleven SNPs or genes were nominally associated with survival under an additive model. Only rs117079142 mapping to *UTP23* and *EIF3H* (Hazard Ratio [HR] = 2.79, 95% Confidence Intervals [CI] = 1.70–4.58, *P* = 4.7 × 10^−5^) and rs9924886 mapping to *CDH1* and *CDH3* (HR = 1.24, 95% CI = 1.12–1.38, *P* = 5.2 × 10^−5^) passed the multiple testing threshold under a recessive model. rs117079142 was an eQTL for *UTP23* and rs9924886 for *CDH1*, *CDH3* and *ZFP90*. Decreased *CDH1* expression in CRCs was associated with worse survival (HR = 2.18, 95% CI = 1.3–3.5, *P* = 1.8 × 10^−3^).

**Conclusion:**

rs117079142 and rs9924886 may represent potential prognostic biomarkers for CRC.

## Introduction

Genome-wide association studies (GWAS) have identified single-nucleotide polymorphisms (SNPs) associated with risk of developing colorectal cancer (CRC) [1]. Some studies have suggested that a subset of these may also influence patient survival [2–7] although other studies have not supported these observations [8–11]. We previously studied the relationship between SNP genotype and patient outcome for 83 CRC-risk SNPs [12] by analysing patients with advanced CRC from the COIN and COIN-B clinical trials [13, 14]. A recent meta-analysis of all available GWAS augmented by transcriptome and methylome-wide association studies (TWAS and MWAS, respectively) has identified further loci taking the total number of CRC-risk loci to 258 [15].

To gain a more comprehensive understanding of the relationship between inherited genetic variation and patient survival, we assessed 233 of these risk loci for their prognostic role in 1926 patients from COIN and COIN-B.

## Materials and methods

### Patients and genotyping

Germline DNAs were extracted from EDTA venous blood samples from 2244 unrelated patients with metastatic or locally advanced colorectal adenocarcinoma participating in the MRC clinical trials COIN (NCT00182715) [13] and COIN-B (NCT00640081) [14]. All patients gave fully informed consent for bowel cancer research (approved by NHS Research Ethics Committee [04/MRE06/60]). COIN patients were randomised 1:1:1 to receive continuous oxaliplatin and fluoropyrimidine chemotherapy, continuous chemotherapy and cetuximab, or intermittent chemotherapy. COIN-B patients were randomised 1:1 to receive intermittent chemotherapy and cetuximab, or intermittent chemotherapy and continuous cetuximab. There was no heterogeneity in overall survival (OS; time from trial randomisation to death or end of trial) between patients when analysed by trial, trial arm, type of chemotherapy received, or cetuximab use [12], so we combined groups for survival analyses. Patient DNAs were genotyped using Affymetrix Axiom Genome-Wide CEU 1 Human Mapping Arrays [16].

Prediction of untyped SNPs was carried out using IMPUTE2 v2.3.0 [17] based on data from the 1000 Genomes Project as reference [18, 19]. After quality control (QC), SNP genotypes were available on 1950 patients. Two patients had no data on survival and a further 22 lacked clinicopathological data leaving 1926 for analysis (of which 1435 died at censorship).

### SNPs and genes analysed

For the 205 CRC-risk SNPs, 19 were directly genotyped, 162 were imputed and 24 were not analysed (one because it was on the X-chromosome which was not genotyped, 19 had INFO scores <0.7 and 4 had minor allele frequencies [MAFs] <0.01). Therefore, in total, 181 CRC-risk SNPs were tested for an association with OS.

For the CRC-risk genes identified from TWAS and MWAS, we used data from a GWAS of COIN and COIN-B (2.9 million SNPs post-QC; [16]). SNPs were mapped to a region spanning 35 kilobases before and 10 kilobases after the transcription zone and analysed using MAGMA v1.07b [20]. Of the 53 genes, 52 were successfully analysed (one had insufficient SNPs in their annotation window).

### Statistical analysis

The relationship between genotype and OS was determined using an additive Cox survival model adjusting for 11 prognostic covariates previously identified in COIN and COIN-B: WHO performance status (*P* = 3.1 × 10^−23^), resection status of the primary tumour (*P* = 1.8 × 10^−21^), WBC count (*P* = 1.2 × 10^−31^), platelet count (*P* = 1.7 × 10^−29^), alkaline phosphatase levels (*P* = 1.5 × 10^−27^), number of metastatic sites (*P* = 1.7 × 10^−13^), liver metastases (*P* = 1.3 × 10^−4^), site of primary tumour (*P* = 9.1 × 10^−9^), surface area of primary tumour (*P* = 1.1 × 10^−5^), time from diagnosis to metastases (*P* = 1.7 × 10^−7^), and metachronous versus synchronous metastases (*P* = 6.0 × 10^−8^) [21]. For gene level analysis in MAGMA, SNP *P*-values were assessed with the linkage disequilibrium (LD) between them using the *multi=snp-wise* option. This model takes advantage of the sum of the -log_10_(*P*) for all SNPs, as well as the top SNP associations within each gene, to assess the association of their constituent genes. For any SNPs or genes nominally associated with OS (*P* < 0.05), we also considered a recessive model to uncover associations potentially missed under additive analyses [22]. We used Bonferroni correction to address multiple testing with *P* < 2.1 × 10^−4^ being considered statistically significant (0.05/233 SNPs or genes tested). Based on the number of patients analysed, our analysis provided over 70% power to demonstrate a HR of 1.2 for SNPs with MAFs >0.30. Power was calculated using the ‘survSNP.power.table’ function from the ‘survSNP’ package in R [23].

### Bioinformatic analyses

We queried the GTEx [24] database to examine SNPs as potential expression quantitative trait loci (eQTLs) for neighbouring genes. Significance for tissue association was set at *P* < 1.0 × 10^-3^ (Bonferroni correction for 49 tissues [0.05/49]). We correlated gene expression with survival by analysing tumours from 597 patients with CRC from The Human Protein Atlas (THPA) [25]. RNA-seq data was reported as median number of fragments per kilobase of exon per million reads (FPKM) [26]. Samples were classified as high expression using the thresholds recommended by THPA (for *CDH1* FPKM was >137; https://www.proteinatlas.org/ENSG00000039068-CDH1/pathology/colorectal+cancer). A log-rank *P*-value was obtained for a difference in survival between patients with CRCs with high and low expression levels. We also performed survival analysis using a linear Cox-proportional hazards model.

## Results

In total, we had survival, clinicopathological and germline genotyping data on 1926 patients with advanced CRC (Table 1). We found that eight CRC-risk SNPs (rs13086367 at 3q13.2, rs280097 at 4q22.2, rs16892766 at 8q23.3, rs117079142 at 8q24.11, rs11255841 at 10p14, rs4444073 at 11p15.4, rs1497077 at 14q22.1 and rs9924886 at 16q22.1) and three CRC-risk genes (*EPB41L2*, *ADAMTS15* and *F2*), were nominally associated with survival under an additive model (Table 2, Supplementary Table 1).

**Table 1:** Clinicopathological features of patients with advanced colorectal cancer.

Clinicopathological factor		Patients with advanced CRC	
		(*n* = 1926)	
		*n*	%
Sex	Male	1261	65.5
	Female	665	34.5
Age	Median (years)	64	-
Overall survival	Median	494	-
	(95% CI) (days)	(469–514)	
WHO performance status	0	900	46.7
	1	885	46.0
	2	141	7.3
Site of primary tumour	Left colon	493	25.6
	Right colon	514	26.7
	Rectosigmoid junction	283	14.7
	Rectum	609	31.6
	Unknown colon	6	0.3
	Multiple sites	21	1.1
Status of primary tumour	Resected	1022	53.1
	Unresected	904	46.9
Stage	1	0	0.0
	2	0	0.0
	3	0	0.0
	4	1926	100.0
Timing of metastases	Metachronous	575	29.9
	Synchronous	1351	70.1
Type of metastases	Liver only	426	22.1
	Liver + others	1019	52.9
	Non-liver^a^	479	24.9
	None	2	0.1
Number of metastatic sites	1	690	35.8
	2	758	39.4
	≥3	478	24.8

Data are *n* (%) or median.

^a^Non-liver metatases included those in the lungs, peritoneum and lymph nodes.

**Table 2:** CRC-risk SNPs or genes associated with survival.

SNP/Gene	Locus	Minor allele	Genes	Additive model			Recessive model		
				HR	95% CI	*P*	HR	95% CI	*P*
rs11255841	10p14	A	*RNA5SP299*	0.88	0.81–0.95	1.7 × 10^−3^	0.85	0.77–0.94	2.0 × 10^−3^
rs16892766	8q23.3	C	*EIF3H, LOC105375713*	1.2	1.06–1.36	4.0 × 10^−3^	1.28	0.95–1.73	0.1
rs117079142	8q24.11	A	*EIF3H, UTP23*	1.26	1.07–1.48	6.0 × 10^−3^	2.79	1.70–4.58	**4.7** **×** **10**^**−5**^
rs4444073	11p15.4	C	*CAND1.11, ADM, LOC653503, SBF2*	1.11	1.03–1.20	7.0 × 10^−3^	1.01	0.94–1.07	0.87
rs9924886	16q22.1	C	*CDH3, CDH1, HSPE1P5, RNA5SP429*	1.12	1.03–1.23	1.1 × 10^−2^	1.24	1.12–1.38	**5.2** **×** **10**^**−5**^
rs280097	4q22.2	C		1.1	1.02–1.19	1.4 × 10^−2^	1.08	1.01–1.15	3.5 × 10^−2^
rs1497077	14q22.1	T	*NID2, RTRAF*	1.1	1.02–1.19	1.7 × 10^−2^	1.12	1.03–1.21	7.2 × 10^−3^
rs13086367	3q13.2	G	*BOC, LINC02044*	0.92	0.86–1.00	4.4 × 10^−2^	0.95	0.89–1.01	0.12
*EPB41L2*	6q23.2	-	-	-	-	2.6 × 10^−3^	-	-	-
*ADAMTS15*	11q24.3	-	-	-	-	1.7 × 10^−2^	-	-	-
*F2*	11p11.2	-	-	-	-	3.2 × 10^−2^	-	-	-

Risk SNPs or genes nominally associated with survival (*P* < 0.05) under an additive model. Genes annotated within a region spanning 50 kb up or downstream of the SNP. Only rs117079142 and rs9924886 passed the threshold for multiple testing (*P* < 2.1 × 10^−4^) when considered under a recessive model (in bold).

**Fig. 1:**
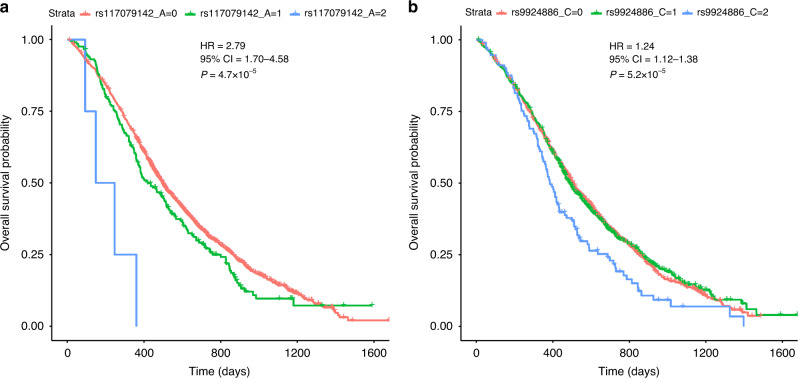
Relationship between rs117079142 and rs9924886 genotype and overall survival. Kaplan–Meier Plots for **a** rs117079142 and **b** rs9924886. *P*-values are for multivariate recessive Cox-regression models and patients are grouped by number of copies of the minor allele. The relationship between genotype and overall survival was adjusted for eleven prognostic covariates: WHO performance status, resection status of the primary tumour, white blood cell count, platelet count, alkaline phosphatase levels, number of metastatic sites, liver metastases, site of primary tumour, surface area of primary tumour, time from diagnosis to metastases and metachronous versus synchronous metastases.

Only rs117079142 (MAF = 0.06, HR = 2.79, 95% CI = 1.70–4.58, *P* = 4.7 × 10^−5^) and rs9924886 (MAF = 0.25, HR = 1.24, 95% CI = 1.12–1.38, *P* = 5.2 × 10^−5^) passed the threshold for multiple testing when considered under a recessive model (Table 2). Patients homozygous for the rs117079142 minor allele (*n* = 4) had a median survival of 198 days compared to 420 days for heterozygotes (*n* = 204) and 497 days for patients homozygous for the major allele (*n* = 1724) (Fig. 1). Patients homozygous for the rs9924886 minor allele (*n* = 113) had a median survival of 385 days compared to 487 days for heterozygotes (*n* = 715) and 507 days for patients homozygous for the major allele (*n* = 1026) (Fig. 1).

rs117079142 was an eQTL for *UTP23* (Supplementary Table 2) and rs9924886 was an eQTL for *CDH1*, *CDH3* and *ZFP90* (Supplementary Table 2) in multiple tissues, but neither were significant in the sigmoid or transverse colon. Low *CDH1* expression in CRCs was associated with worse survival in patients from THPA (5-year survival: low *CDH1* expression = 58%, high *CDH1* expression = 71%, HR = 2.18, 95% CI = 1.3–3.5, *P* = 1.8 × 10^−3^; linear Cox-proportional hazards model *P* = 2.8 × 10^−2^). *UTP23*, *EIF3H* and *CDH3* expression levels were not associated with survival.

## Discussion

In this study, we investigated the relationship between CRC-risk variants and patient outcome. We identified two SNPs associated with survival under a recessive model that were significant beyond the threshold for multiple testing. Interestingly, both SNPs were only nominally significant under additive analyses and others have previously reported on the value of considering recessive models to uncover associations potentially missed [22]. rs117079142 had a modest effect size (HR = 2.79), but relatively low frequency in our cohort; furthermore, in the 1000 genomes dataset the MAF ranges from 0.0076 in the African population to 0.073 in South Asians. In contrast, rs9924886 was more commonly observed in our cohort (and was 0.178 in the African population and 0.3095 in East Asians), but the effect size was lower. These data suggest that neither SNPs are likely to have a direct clinical impact although their identification helps inform potential therapeutic targets.

rs117079142 lies 4 kb downstream of *UTP23*. *UTP23* codes for part of the 90S pre-ribosome and is required for 18S rRNA early processing. Reduced *UTP23* expression has been associated with poor prognosis in patients with ovarian cancer possibly by affecting sensitivity to paclitaxel-based chemotherapy [27]. rs117079142 also lies 23 kb downstream of *EIF3H*, which regulates translation through its interaction with the 40S ribosome and other initiation factors. EIF3 subunits are thought to have oncogenic potential [28] through increased protein synthesis of oncoproteins such as cyclinD1, c-Myc, FGF2 and ornithine decarboxylase [29].

rs9924886 in *CDH3* is a strong proxy for rs9929218 (*D*’ = 0.95 and *r*^2^ = 0.80) and rs9939049 (*D*’ = 0.96 and *r*^2^ = 0.80) in *CDH1* (encoding E-cadherin) that we previously identified as a prognostic biomarker in CRC [12, 30]. Others have also demonstrated a relationship between rs9929218 and survival in CRC patients from Korea [31] and Spain [5]. rs9924886, rs9929218 and rs9939049 are in strong LD with rs16260 [32] in the *CDH1* promoter, which down-regulates *CDH1* expression [33]. Patients homozygous for the minor alleles of these variants would be expected to have reduced E-cadherin expression. Mechanistically, our data are consistent with the downregulation of *CDH1* affecting survival. First, we found that patients homozygous for the rs9924886 minor allele had worse survival and second, we observed that patients with low *CDH1* expression in their colorectal tumours had worse outcome. E-cadherin functions as a transmembrane glycoprotein involved in intercellular adhesion, cell polarity and tissue morphology and regeneration [34], and its loss is a key feature of epithelial to mesenchymal transition during metastasis. Together, these data support a prognostic role for *CDH1* in colorectal tumourigenesis.

rs10161980 has been previously associated with survival from CRC under a recessive model [22]. However, we failed to replicate this SNP in COIN and COIN-B despite having over 98% power. rs10161980 may therefore represent a false-positive or a prognostic biomarker that is specific to patients with earlier stages of disease (we only considered patients with advanced disease in our analyses).

In conclusion, our work provides support for the importance of germline variation as a determinant of patient outcome. Understanding the biological basis of these relationships provides a focus for future work with the goal of identifying novel therapeutic targets for the treatment of CRC.

### Supplementary information

Supplementary Information

## Data Availability

Additional data are available in the Supplementary Information. Scripts are available at https://github.com/Chris-Wills/Wills_et_al_2023_Survival_SNPs.
