# Supplementary material for: Relationship between 233 colorectal cancer risk loci and survival in 1926 patients with advanced disease
Source: BJC Rep. 2023 Jun 22;1:2. doi: 10.1038/s44276-023-00003-z (PMC11041780; doi:10.1038/s44276-023-00003-z)
Supplement: Supplementary file 1 — Supplementary Information [file 44276_2023_3_MOESM1_ESM.docx]

Supplementary Information

Relationship between 233 colorectal cancer risk loci and survival in 1,926 patients with advanced disease

Christopher Wills, Amy Houseman, Katie Watts, Timothy S. Maughan, David Fisher, Richard S. Houlston, Hannah D. West, Valentina Escott-Price and Jeremy P. Cheadle

**Supplementary Table 1. CRC-risk loci and their influence on survival in 1,926 patients with advanced CRC.**

| **SNP/Gene** | **Locus** | **Minor Allele** | **Frequency** | **Additive Model** | | |
| --- | --- | --- | --- | --- | --- | --- |
|  |  |  |  | **HR** | **95% CI** | ***P*** |
| rs2807367 | 1p36.12 | G | 0.32 | 0.99 | 0.91-1.07 | 0.77 |
| rs34963268 | 1p36.12 | C | 0.17 | 0.94 | 0.85-1.04 | 0.23 |
| rs61776719 | 1p34.3 | C | 0.45 | 1.04 | 0.94-1.14 | 0.44 |
| rs12143541 | 1p32.3 | G | 0.16 | 1.03 | 0.94-1.14 | 0.52 |
| rs7542665 | 1p31.3 | T | 0.32 | 1.01 | 0.93-1.09 | 0.83 |
| rs3124454 | 1p31.1 | T | 0.40 | 1.02 | 0.94-1.10 | 0.67 |
| rs6660031 | 1p13.3 | A | 0.34 | 0.99 | 0.92-1.07 | 0.87 |
| rs5028523 | 1q24.3 | G | 0.45 | 1.03 | 0.96-1.11 | 0.38 |
| rs8179460 | 1q25.3 | C | 0.41 | 1.02 | 0.95-1.10 | 0.60 |
| rs12137232 | 1q32.1 | T | 0.44 | 1.01 | 0.94-1.09 | 0.74 |
| rs12078075 | 1q32.1 | G | 0.08 | 1.10 | 0.96-1.26 | 0.17 |
| rs6691170 | 1q41 | T | 0.39 | 0.93 | 0.86-1.00 | 0.06 |
| rs2078095 | 1q43 | G | 0.28 | 0.97 | 0.89-1.05 | 0.41 |
| rs7606562 | 2p16.3 | A | 0.33 | 1.01 | 0.93-1.10 | 0.75 |
| rs11692435 | 2q11.2 | A | 0.06 | 0.99 | 0.84-1.18 | 0.94 |
| rs1446585 | 2q21.3 | G | 0.23 | 0.94 | 0.86-1.03 | 0.18 |
| rs448513 | 2q24.2 | C | 0.34 | 0.99 | 0.92-1.07 | 0.84 |
| rs4668039 | 2q24.3 | G | 0.18 | 0.96 | 0.87-1.06 | 0.40 |
| rs6434979 | 2q33.1 | A | 0.50 | 1.02 | 0.94-1.09 | 0.68 |
| rs4675253 | 2q33.1 | G | 0.34 | 0.98 | 0.90-1.06 | 0.56 |
| rs3731861 | 2q35 | C | 0.36 | 0.94 | 0.87-1.01 | 0.11 |
| rs1800734 | 3p22.2 | A | 0.21 | 1.07 | 0.97-1.17 | 0.17 |
| rs35470271 | 3p22.1 | G | 0.14 | 1.01 | 0.91-1.13 | 0.82 |
| rs2001732 | 3p21.1 | T | 0.12 | 1.00 | 0.89-1.13 | 0.95 |
| rs2581817 | 3p21.1 | C | 0.42 | 0.96 | 0.89-1.03 | 0.26 |
| rs704417 | 3p14.1 | C | 0.49 | 1.00 | 0.93-1.08 | 0.98 |
| rs7623129 | 3p14.1 | T | 0.43 | 1.00 | 0.93-1.08 | 0.95 |
| rs67550176 | 3p14.1 | C | 0.20 | 0.98 | 0.90-1.08 | 0.69 |
| rs13086367 | 3q13.2 | G | 0.46 | 0.92 | 0.86-1.00 | 4.4x10^-2^ |
| rs12635946 | 3q13.2 | T | 0.37 | 0.99 | 0.92-1.07 | 0.85 |
| rs10049390 | 3q22.2 | G | 0.21 | 1.00 | 0.90-1.11 | 0.97 |
| rs113569514 | 3q22.2 | C | 0.11 | 0.98 | 0.86-1.11 | 0.71 |
| rs10936599 | 3q26.2 | T | 0.23 | 1.00 | 0.92-1.10 | 0.98 |
| rs280097 | 4q22.2 | C | 0.41 | 1.10 | 1.02-1.19 | 1.4x10^-2^ |
| rs2007403 | 4q24 | C | 0.34 | 0.97 | 0.89-1.05 | 0.41 |
| rs2388976 | 4q26 | A | 0.45 | 0.97 | 0.90-1.04 | 0.39 |
| rs11727676 | 4q31.21 | C | 0.11 | 1.03 | 0.91-1.16 | 0.66 |
| rs10006803 | 4q31.3 | C | 0.48 | 1.00 | 0.93-1.08 | 0.92 |
| rs1426947 | 4q34.1 | T | 0.42 | 0.94 | 0.87-1.02 | 0.12 |
| rs55810369 | 5p13.1 | T | 0.23 | 1.00 | 0.91-1.09 | 0.97 |
| rs1445012 | 5p13.1 | C | 0.30 | 1.00 | 0.93-1.09 | 0.94 |
| rs3930345 | 5q14.3 | T | 0.18 | 0.99 | 0.90-1.08 | 0.77 |
| rs12659017 | 5q23.2 | A | 0.24 | 1.02 | 0.94-1.11 | 0.64 |
| rs647161 | 5q31.1 | C | 0.33 | 1.02 | 0.94-1.11 | 0.62 |
| rs1294437 | 6p25.1 | T | 0.33 | 1.02 | 0.94-1.11 | 0.64 |
| rs9379084 | 6p24.3 | A | 0.09 | 1.05 | 0.90-1.22 | 0.53 |
| rs2070699 | 6p24.1 | T | 0.47 | 1.01 | 0.94-1.09 | 0.72 |
| rs209142 | 6p22.1 | C | 0.42 | 1.00 | 0.93-1.07 | 0.90 |
| rs1476570 | 6p22.1 | A | 0.26 | 1.04 | 0.96-1.13 | 0.38 |
| rs116353863 | 6p21.33 | C | 0.01 | 0.79 | 0.56-1.12 | 0.18 |
| rs2517448 | 6p21.33 | T | 0.37 | 1.00 | 0.93-1.08 | 0.98 |
| rs62401893 | 6p21.33 | A | 0.04 | 1.12 | 0.94-1.35 | 0.20 |
| rs2516452 | 6p21.33 | G | 0.11 | 0.95 | 0.84-1.07 | 0.41 |
| rs3830041 | 6p21.32 | T | 0.08 | 1.09 | 0.96-1.25 | 0.20 |
| rs16878812 | 6p21.31 | G | 0.11 | 1.08 | 0.97-1.22 | 0.17 |
| rs9470361 | 6p21.2 | A | 0.24 | 0.98 | 0.89-1.08 | 0.71 |
| rs4711689 | 6p21.1 | G | 0.36 | 0.99 | 0.90-1.09 | 0.83 |
| rs57939401 | 6p21.1 | A | 0.11 | 1.08 | 0.96-1.21 | 0.20 |
| rs2208603 | 6p12.1 | T | 0.29 | 1.06 | 0.97-1.15 | 0.19 |
| rs62404966 | 6p12.1 | T | 0.25 | 1.01 | 0.93-1.11 | 0.76 |
| rs6912214 | 6p12.1 | C | 0.40 | 0.98 | 0.91-1.06 | 0.59 |
| rs6928864 | 6q21 | A | 0.08 | 0.95 | 0.83-1.09 | 0.49 |
| rs145997965 | 6q21 | C | 0.01 | 0.80 | 0.58-1.11 | 0.18 |
| rs6911915 | 6q22.1 | C | 0.43 | 1.02 | 0.95-1.10 | 0.59 |
| rs151127921 | 6q23.2 | T | 0.02 | 0.95 | 0.70-1.31 | 0.77 |
| rs1182197 | 7p22.2 | C | 0.37 | 0.94 | 0.87-1.01 | 0.11 |
| rs7810512 | 7p13 | C | 0.25 | 1.04 | 0.95-1.14 | 0.40 |
| rs80077929 | 7p12.3 | T | 0.12 | 1.02 | 0.91-1.15 | 0.74 |
| rs4236382 | 7p12.3 | G | 0.45 | 0.99 | 0.92-1.07 | 0.86 |
| rs6948177 | 7p12.3 | A | 0.33 | 1.04 | 0.96-1.12 | 0.36 |
| rs12539962 | 7q11.23 | T | 0.20 | 1.00 | 0.90-1.11 | 0.97 |
| rs2527927 | 7q22.1 | A | 0.46 | 1.02 | 0.95-1.10 | 0.56 |
| rs17686932 | 7q32.3 | G | 0.03 | 1.03 | 0.81-1.30 | 0.84 |
| rs60911071 | 8p21.2 | C | 0.02 | 0.95 | 0.74-1.22 | 0.68 |
| rs826732 | 8q12.1 | G | 0.49 | 0.98 | 0.91-1.06 | 0.69 |
| rs2450115 | 8q23.3 | C | 0.15 | 0.98 | 0.88-1.09 | 0.75 |
| rs16892766 | 8q23.3 | C | 0.10 | 1.20 | 1.06-1.36 | 4.0x10^-3^ |
| rs117079142 | 8q24.11 | A | 0.06 | 1.26 | 1.07-1.48 | 6.0x10^-3^ |
| rs6983267 | 8q24.21 | T | 0.45 | 1.07 | 1.00-1.15 | 0.07 |
| rs7013278 | 8q24.21 | T | 0.41 | 0.95 | 0.89-1.03 | 0.23 |
| rs4733767 | 8q24.21 | A | 0.26 | 1.00 | 0.92-1.09 | 0.99 |
| rs7859362 | 9p21.3 | C | 0.48 | 1.04 | 0.96-1.12 | 0.31 |
| rs11557154 | 9p13.3 | T | 0.13 | 1.02 | 0.92-1.14 | 0.71 |
| rs34405347 | 9q22.33 | G | 0.08 | 1.03 | 0.90-1.19 | 0.64 |
| rs10978941 | 9q31.2 | T | 0.19 | 0.99 | 0.90-1.09 | 0.85 |
| rs10817106 | 9q31.3 | C | 0.21 | 1.05 | 0.96-1.16 | 0.26 |
| rs12217641 | 10p14 | T | 0.30 | 1.04 | 0.96-1.12 | 0.40 |
| rs11255841 | 10p14 | A | 0.28 | 0.88 | 0.81-0.95 | 1.7x10^-3^ |
| rs1775910 | 10p12.1 | G | 0.22 | 0.98 | 0.89-1.08 | 0.67 |
| rs1773860 | 10p12.1 | T | 0.49 | 1.04 | 0.97-1.13 | 0.26 |
| rs10821905 | 10q11.23 | A | 0.18 | 1.01 | 0.91-1.11 | 0.89 |
| rs7071258 | 10q23.31 | A | 0.21 | 0.99 | 0.89-1.09 | 0.77 |
| rs6584283 | 10q24.2 | T | 0.47 | 1.01 | 0.93-1.09 | 0.86 |
| rs35564340 | 10q24.2 | A | 0.21 | 1.01 | 0.92-1.10 | 0.89 |
| rs4919687 | 10q24.32 | A | 0.29 | 0.96 | 0.88-1.04 | 0.33 |
| rs12241008 | 10q25.2 | C | 0.10 | 0.99 | 0.86-1.13 | 0.85 |
| rs11196172 | 10q25.2 | A | 0.14 | 0.97 | 0.87-1.08 | 0.61 |
| rs4444073 | 11p15.4 | C | 0.49 | 1.11 | 1.03-1.20 | 7.0x10^-3^ |
| rs174537 | 11q12.2 | T | 0.34 | 1.05 | 0.97-1.13 | 0.26 |
| rs10751097 | 11q13.3 | A | 0.39 | 0.95 | 0.87-1.03 | 0.19 |
| rs11236187 | 11q13.4 | A | 0.47 | 1.01 | 0.94-1.09 | 0.83 |
| rs7946853 | 11q13.4 | T | 0.09 | 1.00 | 0.86-1.16 | 0.99 |
| rs117042741 | 11q13.4 | T | 0.03 | 0.97 | 0.77-1.21 | 0.77 |
| rs55864876 | 11q22.1 | A | 0.08 | 1.00 | 0.87-1.16 | 0.96 |
| rs2155065 | 11q22.1 | C | 0.48 | 0.99 | 0.92-1.06 | 0.75 |
| rs3087967 | 11q23.1 | T | 0.31 | 1.06 | 0.98-1.15 | 0.14 |
| rs497916 | 11q23.3 | T | 0.22 | 1.06 | 0.95-1.18 | 0.31 |
| rs10774214 | 12p13.32 | T | 0.37 | 1.00 | 0.93-1.07 | 0.95 |
| rs3217810 | 12p13.32 | T | 0.09 | 0.93 | 0.80-1.09 | 0.36 |
| rs3217874 | 12p13.32 | T | 0.44 | 0.94 | 0.87-1.02 | 0.13 |
| rs10849432 | 12p13.31 | C | 0.10 | 1.00 | 0.89-1.13 | 1.00 |
| rs10849434 | 12p13.31 | C | 0.26 | 0.98 | 0.90-1.06 | 0.60 |
| rs2730985 | 12q12 | A | 0.49 | 0.95 | 0.88-1.02 | 0.18 |
| rs11169572 | 12q13.12 | C | 0.43 | 1.05 | 0.97-1.13 | 0.20 |
| rs7398375 | 12q13.3 | G | 0.22 | 0.98 | 0.87-1.10 | 0.76 |
| rs7297628 | 12q14.2 | C | 0.47 | 0.98 | 0.90-1.06 | 0.61 |
| rs11178634 | 12q21.1 | T | 0.39 | 0.98 | 0.91-1.06 | 0.64 |
| rs653178 | 12q24.12 | C | 0.46 | 0.93 | 0.87-1.01 | 0.08 |
| rs9634162 | 12q24.21 | G | 0.48 | 0.97 | 0.90-1.05 | 0.52 |
| rs7300312 | 12q24.21 | T | 0.42 | 1.05 | 0.97-1.13 | 0.25 |
| rs7299936 | 12q24.21 | G | 0.42 | 1.02 | 0.94-1.10 | 0.65 |
| rs73208120 | 12q24.22 | G | 0.08 | 1.04 | 0.91-1.19 | 0.55 |
| rs10161980 | 13q13.2 | G | 0.37 | 0.98 | 0.90-1.06 | 0.63 |
| rs12427846 | 13q13.3 | C | 0.26 | 0.99 | 0.91-1.08 | 0.90 |
| rs45597035 | 13q22.1 | G | 0.33 | 1.00 | 0.92-1.09 | 0.97 |
| rs78341008 | 13q22.1 | C | 0.07 | 0.96 | 0.84-1.11 | 0.58 |
| rs1886450 | 13q22.1 | A | 0.29 | 0.96 | 0.88-1.04 | 0.29 |
| rs9318511 | 13q22.3 | A | 0.12 | 0.96 | 0.86-1.08 | 0.53 |
| rs1078563 | 13q34 | G | 0.34 | 0.96 | 0.88-1.04 | 0.30 |
| rs4600332 | 13q34 | A | 0.34 | 1.01 | 0.93-1.10 | 0.77 |
| rs28611105 | 14q22.1 | G | 0.20 | 0.98 | 0.89-1.08 | 0.68 |
| rs1497077 | 14q22.1 | T | 0.34 | 1.10 | 1.02-1.19 | 1.7x10^-2^ |
| rs1951864 | 14q22.2 | A | 0.38 | 1.05 | 0.97-1.13 | 0.26 |
| rs35107139 | 14q22.2 | C | 0.37 | 1.01 | 0.92-1.10 | 0.83 |
| rs4901473 | 14q22.2 | G | 0.39 | 1.02 | 0.94-1.10 | 0.62 |
| rs17094983 | 14q23.1 | A | 0.11 | 1.02 | 0.91-1.15 | 0.76 |
| rs8020436 | 14q23.1 | A | 0.41 | 0.98 | 0.91-1.06 | 0.68 |
| rs80158569 | 14q32.2 | A | 0.05 | 0.95 | 0.80-1.12 | 0.54 |
| rs1554865 | 15q13.3 | C | 0.23 | 1.01 | 0.93-1.10 | 0.77 |
| rs1919364 | 15q13.3 | C | 0.49 | 1.00 | 0.93-1.08 | 0.99 |
| rs17816465 | 15q13.3 | A | 0.21 | 1.06 | 0.97-1.17 | 0.20 |
| rs3809570 | 15q22.31 | A | 0.17 | 1.06 | 0.94-1.20 | 0.31 |
| rs56324967 | 15q22.33 | T | 0.29 | 1.02 | 0.93-1.12 | 0.62 |
| rs77148098 | 15q23 | A | 0.10 | 1.00 | 0.89-1.13 | 0.94 |
| rs8031386 | 15q23 | A | 0.25 | 0.96 | 0.88-1.04 | 0.30 |
| rs71407320 | 15q26.1 | T | 0.09 | 0.92 | 0.81-1.05 | 0.20 |
| rs9924886 | 16q22.1 | C | 0.25 | 1.12 | 1.03-1.23 | 1.1x10^-2^ |
| rs3936188 | 16q23.2 | A | 0.44 | 0.98 | 0.90-1.05 | 0.52 |
| rs847208 | 16q24.1 | C | 0.34 | 1.03 | 0.94-1.14 | 0.49 |
| rs7206216 | 16q24.1 | A | 0.49 | 0.99 | 0.92-1.08 | 0.89 |
| rs62042090 | 16q24.1 | T | 0.21 | 1.04 | 0.95-1.15 | 0.40 |
| rs12603526 | 17p13.3 | C | 0.02 | 0.99 | 0.76-1.30 | 0.96 |
| rs4968127 | 17p13.3 | G | 0.39 | 1.01 | 0.93-1.09 | 0.88 |
| rs11247566 | 17p13.3 | A | 0.43 | 1.08 | 0.97-1.20 | 0.18 |
| rs1078643 | 17p12 | G | 0.18 | 0.94 | 0.84-1.05 | 0.28 |
| rs983318 | 17q24.3 | A | 0.22 | 1.05 | 0.95-1.16 | 0.31 |
| rs1791373 | 18p11.31 | T | 0.46 | 1.04 | 0.97-1.12 | 0.26 |
| rs2337113 | 18q21.1 | G | 0.43 | 0.99 | 0.92-1.07 | 0.88 |
| rs34797592 | 19p13.11 | T | 0.06 | 0.96 | 0.80-1.14 | 0.64 |
| rs28840750 | 19q13.11 | G | 0.03 | 0.99 | 0.80-1.23 | 0.94 |
| rs1800469 | 19q13.2 | A | 0.28 | 0.97 | 0.89-1.06 | 0.49 |
| rs12979278 | 19q13.33 | C | 0.43 | 1.00 | 0.92-1.08 | 0.99 |
| rs11670192 | 19q13.43 | A | 0.19 | 0.95 | 0.86-1.06 | 0.36 |
| rs966816 | 20p12.3 | G | 0.36 | 0.97 | 0.90-1.05 | 0.46 |
| rs994308 | 20p12.3 | T | 0.38 | 1.02 | 0.94-1.10 | 0.66 |
| rs4813802 | 20p12.3 | G | 0.38 | 0.99 | 0.91-1.06 | 0.71 |
| rs28488 | 20p12.3 | C | 0.36 | 0.98 | 0.90-1.06 | 0.58 |
| rs2423279 | 20p12.3 | C | 0.22 | 1.04 | 0.93-1.16 | 0.49 |
| rs6059938 | 20q11.22 | A | 0.47 | 0.97 | 0.90-1.04 | 0.37 |
| rs6065668 | 20q13.12 | T | 0.22 | 1.02 | 0.91-1.14 | 0.76 |
| rs6017248 | 20q13.12 | A | 0.25 | 0.96 | 0.88-1.05 | 0.40 |
| rs6066825 | 20q13.13 | G | 0.34 | 0.97 | 0.89-1.04 | 0.37 |
| rs6012915 | 20q13.13 | T | 0.43 | 1.07 | 0.99-1.15 | 0.10 |
| rs6095946 | 20q13.13 | T | 0.35 | 1.07 | 0.97-1.17 | 0.20 |
| rs6091189 | 20q13.13 | T | 0.12 | 1.08 | 0.95-1.22 | 0.26 |
| rs6014965 | 20q13.31 | G | 0.45 | 1.01 | 0.93-1.09 | 0.88 |
| rs13831 | 20q13.32 | A | 0.30 | 1.01 | 0.93-1.09 | 0.79 |
| rs9983528 | 21q22.3 | A | 0.12 | 0.98 | 0.87-1.10 | 0.72 |
| rs4616575 | 22q12.1 | G | 0.49 | 1.02 | 0.94-1.10 | 0.66 |
| rs34256596 | 22q13.2 | A | 0.22 | 0.98 | 0.89-1.08 | 0.69 |
| rs9614460 | 22q13.31 | G | 0.34 | 0.99 | 0.91-1.07 | 0.73 |
| *EPB41L2* | 6q23.2 | - | - | - | - | 2.6x10^-3^ |
| *ADAMTS15* | 11q24.3 | - | - | - | - | 1.7x10^-2^ |
| *F2* | 11p11.2 | - | - | - | - | 3.2x10^-2^ |
| *LEMD3* | 12q14.3 | - | - | - | - | 0.05 |
| *CSRNP1* | 3p22.2 | - | - | - | - | 0.07 |
| *TAGLN* | 11q23.3 | - | - | - | - | 0.07 |
| *SMAD4* | 18q21.2 | - | - | - | - | 0.09 |
| *LINGO4* | 1q21.3 | - | - | - | - | 0.13 |
| *CCDC183* | 9q34.3 | - | - | - | - | 0.15 |
| *CLIP1* | 12q24.31 | - | - | - | - | 0.16 |
| *PGAP3* | 17q12 | - | - | - | - | 0.18 |
| *ERBB2* | 17q12 | - | - | - | - | 0.18 |
| *SPSB1* | 1p36.22 | - | - | - | - | 0.19 |
| *ANO7P1* | 1p36.13 | - | - | - | - | 0.21 |
| *ARHGEF4* | 2q21.1 | - | - | - | - | 0.23 |
| *BECN1* | 17q21.31 | - | - | - | - | 0.23 |
| *DIRC2* | 3q21.1 | - | - | - | - | 0.23 |
| *NRBP1* | 2p23.3 | - | - | - | - | 0.24 |
| *PCSK7* | 11q23.3 | - | - | - | - | 0.24 |
| *C15orf39* | 15q24.2 | - | - | - | - | 0.26 |
| *LMX1B* | 9q33.3 | - | - | - | - | 0.26 |
| *KBTBD4* | 11p11.2 | - | - | - | - | 0.28 |
| *ATP8B1* | 18q21.31 | - | - | - | - | 0.28 |
| *GAB1* | 4q31.21 | - | - | - | - | 0.36 |
| *LIF* | 22q12.2 | - | - | - | - | 0.39 |
| *FBLN7* | 2q13 | - | - | - | - | 0.40 |
| *CDK6* | 7q21.2 | - | - | - | - | 0.40 |
| *LLGL1* | 17p11.2 | - | - | - | - | 0.44 |
| *RHOG* | 11p15.4 | - | - | - | - | 0.47 |
| *SIPA1* | 11q13.1 | - | - | - | - | 0.48 |
| *SETBP1* | 18q12.3 | - | - | - | - | 0.50 |
| *FAM98A* | 2p22.3 | - | - | - | - | 0.51 |
| *TMEM170A* | 16q23.1 | - | - | - | - | 0.53 |
| *CDKN2AIPNL* | 5q31.1 | - | - | - | - | 0.58 |
| *PSMD13* | 11p15.5 | - | - | - | - | 0.62 |
| *ATP2C2* | 16q24.1 | - | - | - | - | 0.62 |
| *GJA4* | 1p34.3 | - | - | - | - | 0.64 |
| *TOX4* | 14q11.2 | - | - | - | - | 0.65 |
| *RPL5* | 1p22.1 | - | - | - | - | 0.75 |
| *ARHGEF19* | 1p36.13 | - | - | - | - | 0.76 |
| *PSMC3IP* | 17q21.2 | - | - | - | - | 0.77 |
| *PLA2G15* | 16q22.1 | - | - | - | - | 0.77 |
| *PLEKHG6* | 12p13.31 | - | - | - | - | 0.79 |
| *GBE1* | 3p12.2 | - | - | - | - | 0.80 |
| *PSMC5* | 17q23.3 | - | - | - | - | 0.81 |
| *CDKAL1* | 6p22.3 | - | - | - | - | 0.85 |
| *FBXO38* | 5q32 | - | - | - | - | 0.87 |
| *ME3* | 11q14.2 | - | - | - | - | 0.87 |
| *OR51E2* | 11p15.4 | - | - | - | - | 0.87 |
| *BNIP2* | 15q22.2 | - | - | - | - | 0.90 |
| *ACP6* | 1q21.2 | - | - | - | - | 0.91 |
| *AXIN1* | 16p13.3 | - | - | - | - | 0.99 |

**Supplementary Table 2 – Relationship between rs117079142 and rs9924886 and gene expression.**

| **SNP** | **Gene** | **Tissue** | ***P*** |
| --- | --- | --- | --- |
| rs117079142 | *UTP23* | Brain - Cortex | 1.1x10^-6^ |
|  |  | Brain - Cerebellum | 1.9x10^-6^ |
|  |  | Brain - Cerebellar Hemisphere | 5.5x10^-6^ |
|  |  | Muscle – Skeletal | 3.1x10^-5^ |
|  |  |  |  |
| rs9924886 | *CDH1* | Brain - Caudate (basal ganglia) | 2.4x10^-11^ |
|  |  | Spleen | 1.5x10^-10^ |
|  |  | Nerve - Tibial | 1.9x10^-9^ |
|  |  | Brain - Putamen (basal ganglia) | 5.7x10^-8^ |
|  |  | Oesophagus - Mucosa | 2.1x10^-7^ |
|  |  | Brain - Hippocampus | 9.3x10^-6^ |
|  |  | Brain - Substantia nigra | 2.1x10^-5^ |
|  |  | Pituitary | 1.1x10^-4^ |
|  |  |  |  |
|  | *CDH3* | Brain - Cortex | 2.0x10^-6^ |
|  |  | Pituitary | 1.2x10^-4^ |
|  |  |  |  |
|  | *ZFP90* | Whole Blood | 1.1x10^-28^ |
|  |  | Lung | 1.3x10^-11^ |
|  |  | Muscle - Skeletal | 6.5x10^-9^ |
|  |  | Spleen | 1.0x10^-6^ |
|  |  | Cells - EBV-transformed lymphocytes | 3.5x10^-5^ |
|  |  | Adipose - Visceral (Omentum) | 1.0x10^-4^ |
